# Extracorporeal shock wave lithotripsy: Prematurely falling out of favour? A 7 year retrospective study from an Australian high‐volume centre

**DOI:** 10.1002/bco2.314

**Published:** 2023-12-02

**Authors:** Nishal Patel, Brittany Stephenson‐Smith, Jay Roberts, Akshay Kothari

**Affiliations:** ^1^ Department of Urology The Prince Charles Hospital Brisbane Queensland Australia; ^2^ Faculty of Medicine The University of Queensland Brisbane Queensland Australia; ^3^ Department of Urology Royal Brisbane and Women's Hospital Brisbane Queensland Australia

**Keywords:** ESWL/surgery, retrospective case series, urolithiasis/surgery

## Abstract

**Objectives:**

The aim of this study is to audit 7 years of data with a 3 year follow up from a high‐volume stone centre performing extracorporeal shock wave lithotripsy (ESWL) to evaluate efficacy in stone clearance compared to existing knowledge and understand reasons for this performance.

**Methods:**

Patients who received ESWL treatment for renal or proximal ureteric stones at a single centre between January 2012 and January 2019 (to allow minimum 3 year follow up) were retrieved. A retrospective analysis was performed cross referencing for stone size, location, treatment and need for further procedures. Ethical approval was granted through Metro North HHS HREC, Queensland, Australia.

**Results:**

A total of 1930 patients met inclusion criteria. Fifty‐seven percent (*n* = 1100) underwent left‐sided ESWL, compared to 43% (*n* = 830) on the right. Stone size and location were both statistically significant to treatment outcome. Small stones (<1 cm) had an overall clearance rate of 81.9%, medium stones (1–2 cm) had a clearance rate of 60.6% and stones (>2 cm) had a clearance rate of 31.3%. Small stones in an upper calyx had the highest clearance rate (87.5%, *n* = 120). Allowing for two procedures, 89% of stones were treated successfully.

**Conclusion:**

ESWL remains a legitimate option for the treatment of small and medium sized renal calculi. ESWL stone clearance rates at our centre are higher than published elsewhere and serve as proof to its efficacy. X‐ray imaging on the day of the procedure, heavy consultant input and frequent intra‐operative imaging are cited as key reasons for success. Further research is warranted to elucidate factors affecting stone clearance rate and to enable more standardised outcomes. Further investment may be required into ESWL provisions in most Australian states and especially in Queensland to enable its continued use in contrast to developing endourological techniques.

## 1 INTRODUCTION

The incidence of renal stone disease is increasing globally leading to a rise in number of overall stone interventions.^1^ This translates to rising economic burden, estimated at $5.3 billion dollars annually in the United States and projected to rise by $1.3 billion dollars annually up until 2030.^2^, ^3^ This increasing prevalence necessitates safe, efficacious and affordable treatment.^3^ Extracorporeal shock wave lithotripsy (ESWL) has long been the mainstay for treatment of small‐ to medium‐sized renal stones; however, its popularity has dramatically decreased in recent years.

**FIGURE 1:**
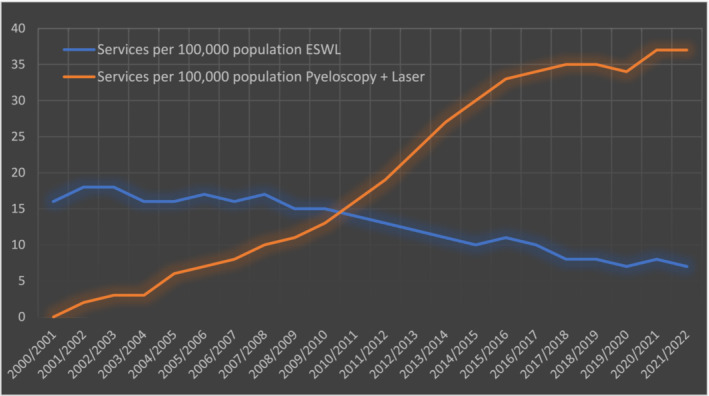
Twenty‐year trend in Australian public renal stone treatment.

An analysis using Australian Medicare Data has shown that ESWL provision per 100 000 population has fallen by 56% from 16 in 2000 to 7 in 2020. In contrast, pyeloscopy and laser services have increased from 1 in 2000 to 37 per 100 000 population in 2020^4^ (Figure 1). Despite this, the cost of ESWL remains significantly cheaper at $730 per procedure compared to $1127 for pyeloscopy.

Recent advances in endoscopic technology have improved intrarenal accessibility. These advances include narrower and more flexible ureteroscopes with improved image resolution as well as their ability to accommodate more sophisticated lasers and baskets to facilitate lithotripsy and stone extraction.^4^ Furthermore, ureteroscopic equipment has become more readily available, making it easier to learn and perform. This likely explains the findings published by Matlaga et al.^5^ who demonstrated a greater use of ureteroscopy among early career Urologists. Similarly, a paper by Bandi et al.^6^ found that urologists who had been practising less than 5 years were more likely to utilise ureteroscopy and percutaneous nephrolithotomy for management of renal stones, than SWL.

Despite the rising favour for endoscopic treatment, the literature has shown that stone clearance rates remain comparable between ESWL and ureteroscopy for small‐ (less than 1 cm) and medium‐ (1 to 2 cm) sized stones in the proximal ureter as well as the kidney.^7^, ^8^

Our study aimed to audit 7 years of data from a high‐volume stone centre performing ESWL to evaluate its efficacy in stone clearance compared to existing knowledge and understand reasons for this performance.

## 2 MATERIALS AND METHODS

### 2.1 Study design

Patients who underwent ESWL at a single centre over a 7 year period between 2012 and 2019 were retrospectively retrieved. Their medical records were then analysed until 2022 to allow minimum of 3 year follow up. Ethical approval was granted through Metro North HHS HREC, Queensland, Australia.

### 2.2 Eligibility Criteria

Patients over the age of 17 who had a renal or proximal ureteric stone defined on noncontrast CT KUB (Kidney–Ureter‐Bladder) which was also visible on XR (X‐Ray) or Ultrasound met inclusion criteria. Patients were excluded from the study if they had recieved an ipsilateral stone procedure within 3 years of enrolment. Furthermore patients were exlcuded due to anatomical factors impacting stone treatment (PUJ obstruction, horse‐shoe kidney and morbid obesity), or contraindications to ESWL such as bleeding diathesis, pacemaker or pregnancy.

### 2.3 Outcome measures and data management

Data was extracted primarily from patient operation notes onto a secure Excel file. Extracted data included patient demographics and medical history and stone factors such as size, location and number. Stone size was divided into three groups: small (<1 cm), medium (1–2 cm) and large (>2 cm). Stone location was subdivided into upper calyx (UC), middle calyx (MC), lower calyx (LC), renal pelvis and proximal ureter. Data pertaining to complications was extracted from Emergency Medicine presentation data.

The primary outcome measure for our study was treatment success. Treatment success was defined as requirement of no further ipsilateral stone procedure within 3 years, and this method was chosen to account for discrepancy in fragment detection between CT and X‐ray.

Our centre offers re‐intervention for residual fragments 4 mm or larger or those deemed to be causing symptoms. Furthermore, our centre routinely performs cystoscopic ureteric stent insertion for all patients with stones ≥15 mm at time of ESWL. Subsequent stent removal is done via flexible cystoscopy under local anaesthetic after their follow‐up review and was not classified as a second stone procedure in this study where further ESWL was not performed.

### 2.4 Statistical methods

Synthesis of collected data was performed using categorical and continuous variables. Chi‐squared test and binary regression and odds ratio (OR) were used to draw inference from available data.

## 3 RESULTS

A total of 1930 patients met inclusion criteria. Fifty‐seven percent (*n* = 1100) underwent left‐sided ESWL, compared to 43% (*n* = 830) on the right.

For any size or location, stone clearance rate was 68.1% (*n* = 1306), with 31.9% (*n* = 611) requiring a further ipsilateral stone procedure within 3 years. Outcome was not clear in 13 patients.

### 3.1 Stone size

Stone size was statistically significant to clearance rate (*χ* ^2^ = 189.83, *df* = 3, *p*‐value < 0.001) (Table 1). Small stones (<1 cm) were treated most (*n* = 884) and had an 81.9% clearance rate in contrast to large stones (>2 cm) that were treated less commonly (*n* = 138) and had a 31% clearance rate. Stones between 1 and 2 cm in size were successfully treated with one procedure 60.6% of the time.

**TABLE 1:** Stone size.

	Failure		Success		Valid	NAs
	*N*	%	*N*	%		
Undefined	195	39.80%	295	60.20%	490	10
Small	160	18.10%	724	81.90%	884	1
Medium	161	39.75%	244	60.25%	405	1
Large	95	68.84%	43	31.16%	138	1
**Sum**	**611**	**31.87%**	**1306**	**68.13%**	**1917**	**13**

*Note*: *χ* ^2^ = 189.83, *df* = 3, *p*‐value < 0.001.

### 3.2 Stone location

Location of stone was also statistically significant (*χ* ^2^ = 23.379, *df* = 6, *p*‐value < 0.001; Table 2) but mattered less than the size. Interestingly, stones in lower calyces had a higher clearance rate overall (70.7%, *n* = 507); however, this result was likely impacted by comparatively large number of small lower calyceal stones treated.

**TABLE 2:** Stone location.

	Failure		Success		Valid	NAs
	*N*	%	*N*	%		
MC	87	29.79%	205	70.21%	292	1
UC	79	30.04%	184	69.96%	263	0
LC	210	29.29%	507	70.71%	717	1
Renal Pelvis	178	35.46%	324	64.54%	502	8
Ureter	87	40.47%	128	59.53%	215	0
Other	12	60.00%	8	40.00%	20	2
Unspecified	52	40.31%	77	59.69%	129	1

*Note*: *χ* ^2^ = 23.379, *df* = 6, *p*‐value < 0.001.

**FIGURE 2:**
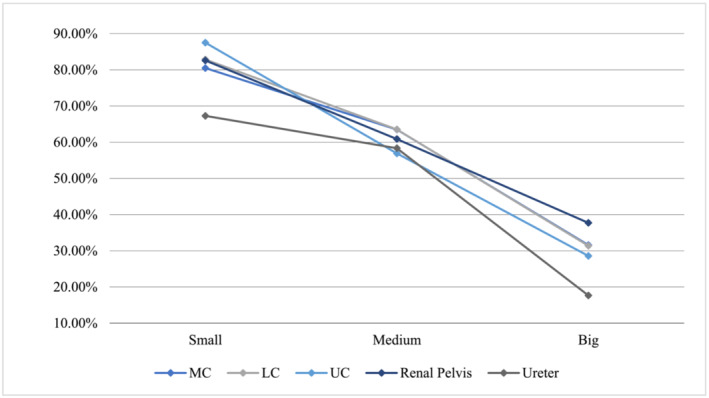
Share of successful kidney stone treatment.

Figure 2 depicts clearance rates for size and location. Small stones in an upper calyx had the highest rate of success (87.5%, *n* = 120). The average success rate for small renal stones was 84.4% (*n* = 806). This figure dropped to 81.9% when incorporating small proximal ureteric stones. For medium‐sized stones, clearance rates were 63.5% in the middle and lower calyces, with an average clearance rate of 61.2% in the kidney (*n* = 417) and 60.6% overall (*n* = 465). Regarding large stones, a 37.7% (*n* = 61) clearance rate was found for stones in the renal pelvis and an average renal stone success rate of 32.3% (*n* = 152).

When allowing for two procedures within 3 years, 89% of stones were treated successfully for any size or location (*n* = 926).

There was no difference in overall stone clearance rate between males (67.8% *n* = 831) and females (68.7% *n* = 475), as shown in Table 3.

**TABLE 3:** Logistic regression model.

	B	Wald	*df*	Sig.	OR	Lower	Upper
Constant	0.878	13.456	1	0.000	2.406		
Multiple	0.385	3.008	1	0.083	1.469	0.951	2.269
Size (Undefined)							
Small	1.113	73.357	1	0.000	3.045	2.360	3.928
Medium	−0.045	0.094	1	0.760	0.956	0.719	1.273
Large	−1.286	34.268	1	0.000	0.276	0.180	0.425
Location (Unspecified)							
MC	−0.148	0.785	1	0.376	0.863	0.622	1.196
UC	0.008	0.002	1	0.965	1.008	0.718	1.415
LC	0.064	0.195	1	0.659	1.066	0.803	1.414
Renal Pelvis	−0.032	0.041	1	0.840	0.968	0.707	1.325
Ureter	−0.553	8.724	1	0.003	0.575	0.398	0.830
Sex (Female)	−0.100	0.820	1	0.365	0.904	0.728	1.124
Age	−0.007	3.367	1	0.067	0.993	0.986	1.000

*Note*: *χ* ^2^ = 209.659, *df* = 11, *p*‐value < 0.001, Nagelkerke *R* ^2^: 0.145.

The logistic regression model is demonstrated in Table S3. Small stones were three times more likely to be treated successfully (OR = 3.02, 95%CI [2.36, 3.92]), whereas large stones or ureteric stones were more likely to require more than one treatment episode. The model also demonstrates that multiple stones adding up to a certain size have a higher treatment success rate than one large stone.

### 3.3 Complications

Overall complication rate was 9% (*n* = 85). Patients with undefined stone characteristics were removed from complication tabulation. A higher complication rate was observed in patients with large stones (18%, *n* = 25) compared to medium (8%, *n* = 33) and small stones (3%, *n* = 27). The most common complications in small‐ and medium‐sized stones collectively were conservatively managed steinstrasse (3%, *n* = 38) and haematuria (2%, *n* = 23). Steinstrasse resulting in operative re‐intervention was deemed as treatment failure. In large stones, haematuria was seen more commonly (13%, *n* = 18), as well as other complications such as urinary frequency, dysuria and pain not attributable to steinstrasse (14%, *n* = 19), which were likely as a result of stent related symptoms. Retroperitoneal haematoma was a complication of one patient in the series. This data is represented in Table 4.

**TABLE 4:** Complications.

	Small (*N* = 884)		Medium (*N* = 405)		Big (*N* = 138)	
	*N*	%	*N*	%	*N*	%
ED presentation	27	3	33	8	25	18
UTI/sepsis	5	0.6	8	2.0	4	2.9
Haematuria	7	0.8	16	4.0	18	13
Steinstrasse	18	2	20	5	2	1
Other	0	‐	0	‐	19	14

## 4 DISCUSSION

This study represents data from the largest stone centre in the state of Queensland and one of the highest volume stone centres in Australia.

Most global urological guidelines for stone treatment still recommend patients be offered either ESWL or endoscopic treatment for small and medium renal stones.^9^, ^10^, ^11^ Our ESWL clearance rates of 84.4% and 63.5%, respectively, support this recommendation and cement this as a good treatment option. Furthermore, reported complications for endoscopic treatment appear higher than ESWL.^12^, ^13^ The noninvasive nature of ESWL and reduced cost per treatment provide further support to the legitimacy of ESWL as a viable option whose practice should not be lost.

Our reported clearance rates are slightly higher than reported elsewhere. This is likely a result of experience through centralisation of services. Furthermore, improved stone localisation may be a factor with patients getting X‐ray imaging on date of booking as well as day of procedure, along with frequent intra‐operative fluoroscopy for continuous targeting. Dual imaging through availability of intraoperative ultrasound as well as fluoroscopy in selected cases aids in stone localisation. Heavy consultant urologist input in the service as well as with each case likely leads to improved outcomes. This centre also performs ESWL under general anaesthesia in contrast to other centres globally. This may provide a benefit in fragmentation rates due to reduced patient movement as well as regulation of respiratory cycle reducing movement of the kidney during treatment, but this needs further evaluation.

Our overall complication rate of 9% is in keeping with that reported in the literature.^12^, ^13^ Despite a significant number of large stones treated with ESWL, this is not the typical practice of our centre, which utilises PCNL and pyeloscopy more commonly in these cases. Ureteric stenting is utilised in these cases to reduce complications such as steinstrasse, and stent‐related representations are likely responsible for the higher incidence of complications seen in this group.

There appears to be state‐based discrepancies regarding treatment trends for urolithiasis in Australia. Victorian ESWL use has remained relatively stable over the past 2 decades, and the divergence between uptake of laser and fall of ESWL occurred much later here (2009) compared to other states (2002–2004).^4^ This may be explained by the denser population seen in Victoria compared to other states and certainly Queensland, where centralisation of services is a much more difficult prospect logistically. ESWL practices are also likely limited by access to equipment. To our knowledge, there are only three publically available ESWL machines in the state, one of which is an independently operated mobile service. This likely leads to logistical difficulties in providing patient services for most centres that do not have an in‐house lithotripter. Resultant treatment delays therefore likely favour the use of endoscopic treatment. Clearly, for ESWL practice to rebound from its current trajectory, further investment into equipment availability is required.

Further work is needed to standardise renal stone treatments in Australia. To manage the anticipated increase in stone patients in the coming years, forward planning needs to consider how management of these patients can be improved. Data from the recent Covid 19 pandemic favoured the use of ESWL over endoscopic procedures because of the possibility of outpatient treatment reducing exposure from hospital admissions and alleviating bed pressures, as well as potential to avoid general anaesthesia.^13^, ^14^ Perhaps this may serve as a push to maintain or ramp up its provision.

This research is not devoid of limitations. Being a single‐centre study, reproducibility elsewhere may be impacted; however, we hope the large reported numbers act to minimise this. The data was collected via operation notes, and not interrogation of patient images themselves, and thus may lead to stone data inaccuracy. We acknowledge the use of treatment success rather than stone clearance as a primary outcome, and this was selected to account for known inferiority of X‐ray detection of small residual fragments over CT.

## AUTHOR CONTRIBUTIONS

**Nishal Patel:** Conceptualisation, methodology, data curation, analysis and writing. **Brittany Stephenson‐Smith:** Data curation and analysis. **Jay Roberts:** Conceptualisation and reviewing. **Akshay Kothari:** Supervision, conceptualisation and reviewing.

## CONFLICT OF INTEREST STATEMENT

The authors declare that they have no known competing financial interests or personal relationships that could have appeared to influence the work reported in this paper.

## Supporting information


**Data S1.** Supporting information.
