# Supplementary material for: Extracorporeal shock wave lithotripsy: Prematurely falling out of favour? A 7 year retrospective study from an Australian high‐volume centre
Source: BJUI Compass. 2023 Dec 2;5(4):460–5. doi: 10.1002/bco2.314 (PMC11019251; doi:10.1002/bco2.314)
Supplement: Supplementary file 1 — Data S1. Supporting information. [file BCO2-5-460-s001.docx]

**Supplementary Tables**

Undefined stone size:

|  | **Failure** | | **Success** | | **Valid** | **NAs** |
| --- | --- | --- | --- | --- | --- | --- |
|  | *N* | *%* | *N* | *%* |  |  |
| MC | 19 | 41.30% | 27 | 58.70% | 46 | 0 |
| LC | 50 | 35.46% | 91 | 64.54% | 141 | 1 |
| UC | 24 | 37.50% | 40 | 62.50% | 64 | 0 |
| Renal Pelvis | 64 | 43.24% | 84 | 56.76% | 148 | 6 |
| Ureter | 17 | 42.50% | 23 | 57.50% | 40 | 0 |
| *χ^2^ = 2.144, df=4, p-value = 0.709* | | | | | | |

Small sized stones:

|  | **Failure** | | **Success** | | **Valid** | **NAs** |
| --- | --- | --- | --- | --- | --- | --- |
|  | *N* | *%* | *N* | *%* |  |  |
| MC | 32 | 19.51% | 132 | 80.49% | 164 | 1 |
| LC | 59 | 17.15% | 285 | 82.85% | 344 | 0 |
| UC | 15 | 12.50% | 105 | 87.50% | 120 | 0 |
| Renal Pelvis | 31 | 17.42% | 147 | 82.58% | 178 | 0 |
| Ureter | 36 | 32.73% | 74 | 67.27% | 110 | 0 |
| *χ^2^ = 17.920, df=4, p-value = 0.001* | | | | | | |

Medium sized stones:

|  | **Failure** | | **Success** | | **Valid** | **NAs** |
| --- | --- | --- | --- | --- | --- | --- |
|  | *N* | *%* | *N* | *%* |  |  |
| MC | 23 | 36.51% | 40 | 63.49% | 63 | 0 |
| LC | 66 | 36.46% | 115 | 63.54% | 181 | 0 |
| UC | 25 | 43.10% | 33 | 56.90% | 58 | 0 |
| Renal Pelvis | 45 | 39.13% | 70 | 60.87% | 115 | 1 |
| Ureter | 20 | 41.67% | 28 | 58.33% | 48 | 0 |
| *χ^2^ = 1.164, df=4, p-value = 0.884* | | | | | | |

Large sized stones:

|  | **Failure** | | **Success** | | **Valid** | **NAs** |
| --- | --- | --- | --- | --- | --- | --- |
|  | *N* | *%* | *N* | *%* |  |  |
| MC | 13 | 68.42% | 6 | 31.58% | 19 | 0 |
| LC | 35 | 68.63% | 16 | 31.37% | 51 | 0 |
| UC | 15 | 71.43% | 6 | 28.57% | 21 | 0 |
| Renal Pelvis | 38 | 62.30% | 23 | 37.70% | 61 | 1 |
| Ureter | 14 | 82.35% | 3 | 17.65% | 17 | 0 |
| *χ^2^ = 2.647, df=4, p-value = 0.618* | | | | | | |
